# In silico identification of novel PKC βII inhibitors: ligand and receptor based pharmacophore modeling, virtual screening, and molecular dynamics study

**DOI:** 10.1186/1758-2946-4-S1-P45

**Published:** 2012-05-01

**Authors:** Baljinder K Grewal, ME Sobhia

**Affiliations:** 1 Department of Pharmacoinformatics, National Institute of Pharmaceutical Education and Research (NIPER), Sector 67, S.A.S Nagar, Punjab 160062, India

## 

The Protein Kinase C *β*II (PKC*β*II) belongs to conventional class of Protein kinase C enzyme and is preferentially activated during diabetic cardiomyopathy [1]. An effective inhibition of PKC*β*II is the potential option to directly treat the diabetic cardiomyopathy. Till date only one selective PKC*β*II inhibitor, ruboxistaurin reached phase III clinical trial for diabetic complications. Thus, there is an urgent need for exploring available chemical space for new PKC*β*II inhibitors. The sequential virtual screening workflow based on ligand and receptor based query was followed to identify novel PKC*β*II inhibitors. Three different strategies were followed for developing the ligand based model by HipHop module implemented in Catalyst, using: (I) three active and six moderately active compounds; (II) 17 active compounds; (III) docked poses of the compounds used in strategy (II). Receptor based query was developed based on the cocrystallised crystal structure of PKC*β*II with 2-methylbisindolylmaleimide (2mBIM) using the Unity module of Sybyl7.1. The best hypotheses from both methods consist of six features *viz.* one hydrogen bond donor (D), two hydrogen bond acceptor (A), two hydrophobic-aromatic (HYD) and one ring aromatic (R). Virtual screening scheme based on these 3D hypotheses identified a few molecules with higher docking score than the existing inhibitors. In addition, comparative molecular dynamics (MD) simulation studies of uncomplexed PKC*β*II and its complexes with 2mBIM, ruboxistaurin and newly identified compounds were performed to analyze the binding mode of the molecules. This study showed that complexed form of PKC*β*II was more stable than uncomplexed one during simulation period, and showed the stable H-bond formation with Glu421, Val423. This reveals the favorable interactions of identified compounds with PKC*β*II.
